# The increase of microRNA-21 during lung fibrosis and its contribution to epithelial-mesenchymal transition in pulmonary epithelial cells

**DOI:** 10.1186/1465-9921-14-95

**Published:** 2013-09-24

**Authors:** Mitsuhiro Yamada, Hiroshi Kubo, Chiharu Ota, Toru Takahashi, Yukiko Tando, Takaya Suzuki, Naoya Fujino, Tomonori Makiguchi, Kiyoshi Takagi, Takashi Suzuki, Masakazu Ichinose

**Affiliations:** 1 Department of Respiratory Medicine, Tohoku University Graduate School of Medicine, 1-1 Seiryoumachi, Aobaku 980-8574, Sendai, Japan; 2 Department of Advanced Preventive Medicine for Infectious Disease, Tohoku University Graduate School of Medicine, 2-1 Seiryoumachi, Aobaku 980-8575, Sendai, Japan; 3 Department of Thoracic Surgery, Institute of Development, Aging and Cancer, Tohoku University, 4-1 Seiryoumachi, Aobaku 980-8575, Sendai, Japan; 4 Department of Pathology and Histotechnology, Tohoku University Graduate School of Medicine, 2-1 Seiryo-machi, Aoba-ku, Sendai 980-8575, Miyagi-ken, Japan

**Keywords:** microRNAs, Epithelial-mesenchymal transition, Pulmonary fibrosis, Alveolar type II cells, Lung single cell separation

## Abstract

**Background:**

The excess and persistent accumulation of fibroblasts due to aberrant tissue repair results in fibrotic diseases such as idiopathic pulmonary fibrosis. Recent reports have revealed significant changes in microRNAs during idiopathic pulmonary fibrosis and evidence in support of a role for microRNAs in myofibroblast differentiation and the epithelial-mesenchymal transition in the context of fibrosis. It has been reported that microRNA-21 is up-regulated in myofibroblasts during fibrosis and promotes transforming growth factor-beta signaling by inhibiting Smad7. However, expression changes in microRNA-21 and the role of microRNA-21 in epithelial-mesenchymal transition during lung fibrosis have not yet been defined.

**Methods:**

Lungs from saline- or bleomycin-treated C57BL/6 J mice and lung specimens from patients with idiopathic pulmonary fibrosis were analyzed. Enzymatic digestions were performed to isolate single lung cells. Lung epithelial cells were isolated by flow cytometric cell sorting. The expression of microRNA-21 was analyzed using both quantitative PCR and in situ hybridization. To induce epithelial-mesenchymal transition in culture, isolated mouse lung alveolar type II cells were cultured on fibronectin-coated chamber slides in the presence of transforming growth factor-β, thus generating conditions that enhance epithelial-mesenchymal transition. To investigate the role of microRNA-21 in epithelial-mesenchymal transition, we transfected cells with a microRNA-21 inhibitor. Total RNA was isolated from the freshly isolated and cultured cells. MicroRNA-21, as well as mRNAs of genes that are markers of alveolar epithelial or mesenchymal cell differentiation, were quantified using quantitative PCR.

**Results:**

The lung epithelial cells isolated from the bleomycin-induced lung fibrosis model system had decreased expression of epithelial marker genes, whereas the expression of mesenchymal marker genes was increased. MicroRNA-21 was significantly upregulated in isolated lung epithelial cells during bleomycin-induced lung fibrosis and human idiopathic pulmonary fibrosis. MicroRNA-21 was also upregulated in the cultured alveolar epithelial cells under the conditions that enhance epithelial-mesenchymal transition. Exogenous administration of a microRNA-21 inhibitor prevented the increased expression of vimentin and alpha-smooth muscle actin in cultured primary mouse alveolar type II cells under culture conditions that induce epithelial-mesenchymal transition.

**Conclusions:**

Our experiments demonstrate that microRNA-21 is increased in lung epithelial cells during lung fibrosis and that it promotes epithelial-mesenchymal transition.

## Background

In normal tissue repair, collagen deposition after inflammation or injury is an indispensable and reversible reaction for wound healing. However, if the tissue injury is massive or repetitive and/or if the repair process itself becomes abnormal and the excessive and persistent accumulation of myofibroblasts and extracellular matrix proteins, including collagen, has occurred, the result is a fibrotic disease such as idiopathic pulmonary fibrosis (IPF).

MicroRNAs are non-coding RNA molecules that consist of 22 nucleotides and function in the transcriptional and post-transcriptional regulation of gene expression [1]. Recent reports have revealed significant changes in microRNAs during both lung fibrosis and human fibrotic lung disease. Moreover, accumulating evidence suggests a role for microRNAs in myofibroblast differentiation and the endothelial-mesenchymal transition (EMT) in the context of lung fibrosis. let-7d, which is abundantly expressed in epithelial cells in normal lungs, is down-regulated in the lung tissues of IPF patients [2]. The expression of let-7d is inhibited by transforming growth factor-β (TGF-β) [2], and the downregulation of let-7d promotes EMT in epithelial cells and increased collagen deposition in mouse lungs [2]. The microRNA-200 (miR-200) family can reverse EMT and induce an epithelial phenotype in cancer cells [3-8]. The miR-200 family targets the ZEB factors (ZEB1 and ZEB2) that function as transcriptional repressors. Because ZEB factors induce EMT by suppressing the expression of epithelial marker genes, including E-cadherin [9,10], the overexpression of miR-200 s decreases expression of ZEB factors and induces epithelial differentiation. A recent publication showed that the miR-200 family members inhibit TGF-β-induced EMT in a rat alveolar epithelial cell line [11].

MicroRNA-21 (miR-21) was first identified as an anti-apoptotic factor in glioblastomas [12] and has been described as an oncogenic microRNA targeting many tumor suppressor genes, including *PTEN*[13]. Recently, it has been shown that miR-21 has a role in lung fibrosis. miR-21 is increased in the whole lung of bleomycin-instilled mice [14] as well as in lung biopsy samples of IPF patients [14-16]. miR-21 is induced by TGF-β1 and targets the inhibitory Smad, Smad7 [14]. Thus, miR-21 amplifies TGF-β signaling in a positive feedback fashion. Because TGF-β signaling can induce and maintain EMT, we postulated that miR-21 could contribute to EMT in lung epithelial cells during lung fibrosis, though a previous report showed that increased miR-21 expression was primarily localized to myofibroblasts [14].

To investigate the roles of miR-21 in EMT of lung alveolar epithelial cells during lung fibrosis, we first examined the miR-21 expression changes in lung epithelial cells during bleomycin-induced lung fibrosis. Because lungs consist of various types of cells and because expression patterns of molecules, including microRNAs, differ among these cell types, we developed a flow-cytometric cell sorting method for isolating mouse lung epithelial cells from cells such as mesenchymal and endothelial cells. We also examined changes in the expression of these microRNAs in cultured primary mouse lung alveolar type II epithelial cells under EMT-inducing conditions. In addition, to elucidate the role of miR-21 in EMT, we examined whether inhibition of miR-21 attenuated EMT induced by TGF-β in primary mouse alveolar type II cells.

## Methods

### Animal studies

All animal experiments were approved by the Tohoku University Animal Experiment Ethics Committee and were performed in accordance with the Regulations for Animal Experiments and Related Activities at Tohoku University.

### Patients and preparation of tissue samples

Human lung tissue was obtained from patients who underwent lung resections at the Department of Thoracic Surgery at Tohoku University Hospital (Aobaku, Sendai, Japan) or at Ishinomaki Red Cross Hospital (Hebita, Ishinomaki, Japan). Non-fibrotic lung tissue specimens were obtained from 3 patients who underwent surgery for lung cancer. The specimens were resected from portions distal to the cancerous lesions, and histopathological examination confirmed that these non-fibrotic tissues did not contain any lesions, including those associated with cancer, fibrosis, emphysema or inflammatory changes. Fibrotic lung tissue specimens were obtained from 3 patients who underwent open lung biopsy or lung resection because of lung cancer. The patients were clinically diagnosed as having IPF based on pathological examinations of the fibrotic lesions. This study was approved by the Ethics Committees at Tohoku University School of Medicine and the Ishinomaki Red Cross Hospital. All subjects gave informed consent.

### The animal model of pulmonary fibrosis

Seven- to eight-week-old male C57BL/6 mice were used in our experiments. C57BL/6 mice were purchased from CLEA Japan (Yokohama, Japan). All mice were housed in a specific pathogen-free facility and were maintained under constant temperature (24°C), humidity (40%), and light cycle (8:00 A.M. to 8:00 P.M.) conditions, with food and water provided ad libitum. To induce pulmonary fibrosis, mice were treated intratracheally with bleomycin hydrochloride (Nippon Kayaku, Tokyo, Japan) on day 0 as described in our previous study [17]. Briefly, mice were anesthetized with ketamine via intraperitoneal injection and were then instilled with 0.04 mg of bleomycin hydrochloride in 100 μl of saline through a 27G needle inserted between the cartilaginous rings of the trachea. The lungs were harvested 14 days after instillation for further analyses.

### The preparation of single lung cells from whole lung

Single lung cells from mice were isolated as previously described with some modifications [18]. Briefly, mice received an overdose of inhaled halothane, and their lungs were perfused with PBS via the right ventricles. The PBS-perfused lungs were isolated with other mediastinal organs. The Dispase II solution (Roche Applied Science, Mannheim, Germany; final concentration, 2.0 U/mL) was instilled into the lungs through the trachea, which was then ligated with a silk suture. After incubation at 37°C for 50 min, the lungs were separated from the other mediastinal organs. The lungs were then thoroughly minced and digested in PBS containing 0.1% collagenase (Roche Applied Science) and 0.01% deoxyribonuclease I (Sigma-Aldrich, St. Luis, MO) at 37°C for 20 min. The cells were then suspended in red blood cell lysis buffer to remove red blood cells (Sigma-Aldrich) and were subsequently washed with PBS. The cells were then centrifuged and re-suspended in PBS.

### Flow cytometry and the sorting of lung component cells

We used the following antibodies: Alexa Fluor 647-conjugated anti-mouse EpCAM antibody (clone G8.8, Biolegend, San Diego, CA); phycoerythrin (PE)-conjugated anti-mouse VE-cadherin antibody (clone VECD1, Biolegend); and fluorescein isothiocyanate (FITC)-conjugated anti-mouse CD45 antibody (clone 30-F11, Biolegend). To discriminate between live and dead cells, we used 7-amino actinomycin D (7-AAD; eBioscience, San Diego, CA). The antibodies were incubated with the samples for 30 min at 4°C and the samples were then washed. We re-suspended the cells in 2% FBS/PBS and labeled dead cells with 7-AAD. We sorted live and single-cell-gated subpopulations based on their staining patterns using EpCAM, VE-cadherin and CD45 with a FACS Aria II Cell Sorter and FACS Diva ver 6.1 (BD Biosciences, San Jose, CA). FACS analyses were conducted using FCS Express 3 software (De Novo Software, Los Angeles, CA). The sorted epithelial (CD45^-^, EpCAM^+^ cells), endothelial (CD45^-^, VE-cadherin^+^ cells) and mesenchymal cells (CD45^-^, EpCAM^-^, VE-cadherin^-^ cells) were collected in DMEM/10% FCS/Penicillin/Streptomycin/amphotericin B (Life Technologies, Grand Island, NY) for further analyses.

### Isolation of alveolar epithelial type II cells from human lung tissues

Human lung cells were isolated as previously described [19], and human ATII cells were isolated as previously described with some modifications [20]. Briefly, we used phycoerythrin-conjugated anti-human EpCAM antibody, Alexa Fluor 647-conjugated anti-human T1α antibody, and FITC-conjugated anti-human VE-cadherin antibody. To discriminate between live and dead cells, we used 7-amino actinomycin D. We sorted live and single-cell-gated subpopulations based on their staining patterns for EpCAM, T1α, and VE-cadherin expression using a FACS Aria II Cell Sorter and FACS Diva, version 6.1 (BD Biosciences).

### Total RNA purification and the quantification of microRNAs and mRNAs

Total RNA containing microRNAs was purified from the cells using the miRNeasy Mini Kit (Qiagen, Hilden, Germany). A miScript Reverse Transcription Kit was used for reverse transcription of microRNAs and mRNAs into cDNA. The microRNAs were quantified by real-time PCR using an miScript Primer Assay (Qiagen). mRNAs were also quantified by real-time PCR using a QuantiTect Primer Assay (Qiagen). The specific primer sets for quantification were purchased from Qiagen as follows: MS00001827 for miR-200c; MS00011487 for miR-21; MS00033740 for RNU6B snRNA (a constitutively expressed housekeeping control); QT00121163 for E-cadherin; QT00109424 for Surfactant protein C (SP-C); QT00110467 for VE-cadherin; QT00159670 for Vimentin; QT00140119 for alpha-smooth muscle actin (α-SMA); QT00105385 for ZEB1; QT00148995 for ZEB2 (E-cadherin repressors); QT01658692 for glyceraldehyde-3-phosphate dehydrogenase (GAPDH, a constitutively expressed gene).

### In situ hybridization (ISH)

In situ hybridization was performed according to manufacturer’s protocol provided in the MicroRNA ISH Buffer and Controls Kit (Exiqon, Woburn, MA), with some modifications. Briefly, human lung tissues were inflated with 10% formalin solution and embedded in paraffin. Mouse lung tissues were inflated with 4% paraformaldehyde solution and embedded in paraffin. Slides were deparaffinized and incubated with proteinase-K for 10 min at 37°C and washed with PBS. The hybridization mixture contained 5 nM double-DIG LNA™ microRNA probe for miR-21 or scramble-miR as the negative control (Exiqon) and was applied and hybridized for 1 h at 53°C. Slides were washed in SSC buffer and incubated with RNase solution (20 μg/ml, Wako Pure Chemical Industries, Osaka, Japan) for 30 min at 37°C. Slides were washed in SSC buffers and incubated with blocking solution containing goat serum for 15 min at RT. Sheep anti-digoxigenin-POD (poly) Fab fragments (at 1:500 in antibody diluent, Roche) were applied and incubated overnight at 4°C. Slides were then washed and incubated with biotin-SP-conjugated AffiniPure goat anti-horseradish peroxidase (1:200) at 37°C for 30 min (Jackson ImmunoResearch). Finally, the slides were washed and labeled with Streptavidin-AP (Roche). NBT/BCIP solution (Roche) was used for detection. After washing in water and counter-staining with Nuclear Fast Red, the slides were dehydrated and mounted for observation by microscopy.

### The isolation and culture of murine alveolar type II cells

Murine alveolar type II cells were prepared from 7-week-old male C57BL/6 mice by a modification of the method of Corti et al. [21]. Mice were anesthetized with a ketamine-xylazine-atropine mixture by intraperitoneal injection. The abdominal cavity was opened, and mice were exsanguinated by cutting the inferior vena cava and the left renal artery. The diaphragm was cut, and the chest plate and thymus were removed. With the use of a 23G needle on a 10-ml syringe, the lungs were perfused with 10 ml of 0.9% saline via a right ventricle. The trachea was isolated and cannulated with a 20-gauge iv catheter that was secured by a ligature. Two ml BD Dispase solution (BD Bioscience) was rapidly instilled through the cannula into the trachea followed by 0.5 ml low melting point agarose (Sigma-Aldrich) that was preheated to 45°C. The lungs were immediately covered with ice for 2 min to allow the agarose to solidify. After this incubation, the lungs were removed from the animals and incubated in 1 ml BD dispase for 45 min (25°C) before being placed on ice until the next step. The lungs were subsequently transferred to a 100 mm culture dish containing 10 ml of 25 mM HEPES-buffered DMEM/10% FCS/Penicillin/Streptomycin/amphotericin B (Life Technologies) and 100 U/ml DNase I (Sigma-Aldrich). The lung tissue was gently teased from the bronchi by curved forceps and was then gently swirled for 10 min. The resulting cell suspension was filtered sequentially through 100 μm and 40 μm cell strainers (BD Biosciences). The single lung cells were collected by centrifugation at 1200 rpm for 10 min at 4°C. The cells were re-suspended in PBS with 0.5% BSA and 2 mM EDTA and were then incubated with MACS Mouse CD45 MicroBeads (Miltenyi Biotec, Bergisch Gladbach, Germany) for 15 min at 4°C. The cells were then washed before proceeding to magnetic separation by autoMACS (Miltenyi Biotec) to deplete CD45^+^ cells. The collected CD45^-^ cells were then incubated with an anti-FITC-conjugated anti-mouse EpCAM antibody (Biolegend) for 10 min at 4°C. After washing, the cells were incubated with MACS anti-FITC MicroBeads (Miltenyi Biotec) for 15 min at 4°C. After incubation and washing, the cells were separated magnetically by autoMACS (Miltenyi Biotec) to collect the CD45^-^ EpCAM^+^ cells. The collected cells were assessed by immunostaining for EpCAM and pro SP-C. Greater than 99% of the cells were positive for EpCAM and >96% of cells were typically positive for pro-SP-C. Viability was >95%.

The cells were cultured on chamber slides that were coated with Matrigel-rat tail collagen (70:30 vol/vol) to maintain the mouse type II cell phenotype in vitro [22] or were cultured in human fibronectin pre-coated chamber slides (BD Biosciences) under EMT-inducing culture conditions. The cells were maintained in SAGM (Lonza, Basel, Switzerland) without hydrocortisone and containing 5% charcoal/dextran-treated FBS (Invitrogen, Carlsbad, CA) and 10 ng/ml human recombinant KGF (Rocky Hill, NJ) in a 37°C, 5% CO2 incubator as described. For the EMT-inducing culture conditions, 4 ng/ml human recombinant TGF-β was added to the media. The cells were analyzed on culture day 6.

### The transfection of microRNA mimics and/or inhibitors into murine alveolar type II cells

We transfected synthesized miR-200c (Qiagen) and/or a miR-21 inhibitor composed of single-stranded, modified RNAs that specifically inhibit miR-21 function (miScript miRNA Inhibitor; Qiagen) into isolated mouse alveolar type II cells using HiPerFect Transfection Reagent (Qiagen). Control oligonucleotides (Qiagen) were transfected into negative control samples. To optimize transfection conditions, we used AllStars Hs Cell Death Control siRNA (Qiagen), which targets essential cell survival genes. The transfection efficiency was measured by observation of the level of cell death after the transfection of this siRNA. The transfection conditions that resulted in more than 80% cell death when compared with transfection with control oligonucleotides was used in the experiments. After transfection, the alveolar type II cells were cultured on fibronectin-coated chamber slides with SAGM supplemented with 5% charcoal/dextran-treated FBS, 10 ng/ml KGF and 4 ng/ml TGF-β, conditions that enhance EMT. The transfections of the microRNA and/or the miR-21 inhibitor were repeated on day 3. The cells were harvested on day 6.

### Data presentation and statistical analysis

Unless otherwise noted, all data presented are expressed as the means ± standard error of the means (SEM). Statistical analyses were performed using Statistica software (StatSoft Inc., Tulsa, OK, USA). The data were assessed for significance using an unpaired t-test for comparisons between the two groups or by ANOVA with Scheffé’s post hoc method for multiple comparisons. Statistical significance was defined as *p* < 0.05.

## Results

### The identification and isolation of epithelial, endothelial and mesenchymal cells from mouse lungs

**Figure 1:**
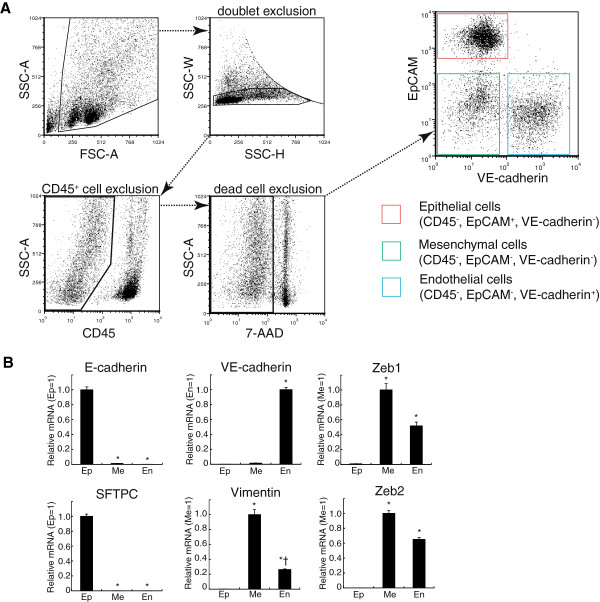
**The identification and isolation of epithelial, endothelial and mesenchymal cells from mouse lungs. (A)** A fluorescence-activated cell sorting strategy. Single lung cells were stained with antibodies against EpCAM, VE-cadherin and CD45. 7-AAD was used for dead cell exclusion. Epithelial, endothelial and mesenchymal cells are identified as CD45^-^ EpCAM^+^ VE-cadherin^-^ cells, CD45^-^ EpCAM^-^ VE-cadherin^+^ cells and CD45^-^ EpCAM^-^ VE-cadherin^-^ cells, respectively. **(B)** The expression of mRNAs in isolated lung epithelial (Ep), mesenchymal (Me) and endothelial (En) cells from untreated mice. The mean and SEM of relative mRNA expression are shown (n = 6). The values relative to those of epithelial cells are presented for E-cadherin and SPC. For VE-cadherin, values relative to those of endothelial cells are presented. For vimentin, ZEB1 and ZEB2, values relative to those of mesenchymal cells are presented. The data were analyzed by one-way analysis of variance with a post hoc test (Scheffé’s test). * p < 0.05 versus lung epithelial cells. † p < 0.05 versus lung mesenchymal cells.

To investigate the levels of expression of miR-21 in lung epithelial cells precisely, we utilized flow cytometric cell sorting to identify and to isolate epithelial, endothelial and mesenchymal cells (Figure 1A). A lung cell suspension was prepared by enzymatic digestion. We first excluded CD45^+^ cells and dead cells by staining with both an anti-CD45 antibody and 7-AAD. Then, CD45^-^ cells were analyzed for surface expression of both EpCAM (a specific marker for epithelial cells) and VE-cadherin (a specific marker for endothelial cells). We identified and isolated three cell populations: CD45^-^ EpCAM^+^ VE-cadherin^-^ cells as epithelial cells; CD45^-^ EpCAM^-^ VE-cadherin^+^ cells as endothelial cells; and mesenchymal cells as CD45^-^ EpCAM^-^ VE-cadherin^-^ cells. The expression of mRNAs of marker genes was examined in these isolated lung cell populations (Figure 1B). E-cadherin (a specific marker for epithelial cells) and surfactant protein-C (SFTPC, a marker for alveolar type II cells) were specifically expressed in isolated lung epithelial cells, whereas VE-cadherin mRNA (a specific marker for endothelial cells) was not detected and the expression levels of Vimentin (a marker for mesenchymal cells) and ZEB1/2 (EMT inducers) were very low when compared to the levels of these markers in lung mesenchymal cells. These data suggested that isolated CD45^-^ EpCAM^+^ VE-cadherin^-^ cells obviously had an epithelial cell phenotype.

### miR-21 increases in lung epithelial cells during experimental bleomycin-induced lung injury

**Figure 2:**
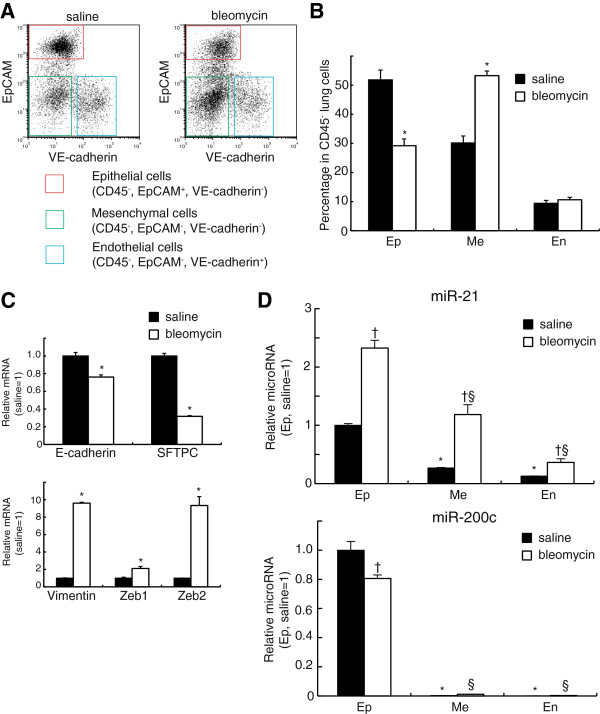
**miR-21 is increased in lung epithelial cells during experimental bleomycin-induced lung injury. (A)** Representative flow cytometric analyses of CD45^-^ lung cells from saline- and bleomycin-treated mice. **(B)** The percentage of each cell type within all CD45^-^ lung cells. Note that bleomycin induced a decrease in the percentage of epithelial cells (Ep) and an increase in that of mesenchymal cells (Me) but no change in endothelial cells (En). **(C)** The relative expression of mRNA of lung epithelial marker genes (E-cadherin and SFTPC) or EMT marker genes (Vimentin, Zeb1 and Zeb2) is shown. **(D)** The relative expression of microRNAs is shown. The values are mean ± SEM (*n* = 6). The values relative to those of the saline-treated epithelial cells are presented. The data were analyzed by Student t test. * p < 0.05 versus epithelial cells in the saline group. † p < 0.05 between saline group and bleomycin group in each cell type. § p < 0.05 versus epithelial cells in the bleomycin group.

To investigate the expression levels of miR-21 in lung epithelial cells during lung fibrosis, we used the mouse model of bleomycin-induced lung fibrosis. Flow cytometric analyses revealed that bleomycin treatment decreased the percentage of epithelial cells and increased the percentage of mesenchymal cells in isolated lung CD45^-^ cells, suggesting that fibrotic changes had occurred in the lungs of bleomycin-treated mice (Figure 2A and 2B). The level of E-cadherin mRNA was significantly decreased in lung epithelial cells that were isolated from bleomycin-treated mice (Figure 2C). SFTPC was also decreased. However, vimentin and ZEB 1/2 were significantly increased in lung epithelial cells that were isolated from bleomycin-treated mice (Figure 2C). These observations implied that epithelial-mesenchymal transition in lung epithelial cells had occurred during experimental bleomycin-induced lung injury.

Next, we examined the changes in expression levels of miR-21 in isolated epithelial, endothelial and mesenchymal cells from lungs treated with saline or bleomycin. In the saline treated mice, miR-21 was expressed in all lung cell populations, but the level of miR-21 in lung epithelial cells was significantly higher compared with that in endothelial or mesenchymal cells. The bleomycin treatment increased miR-21 significantly in all lung cell populations and the levels of miR-21 were also significantly higher in lung epithelial cells when compared with other cells including mesenchymal cells. This result is contrary to previous reports showing that myofibroblasts were the main source of the increased miR-21 levels present in bleomycin-treated lungs (Figure 2D). These observations suggest that miR-21 may also participate in EMT of lung epithelial cells. We also examined the levels of miR-200c, one of the miR-200 family microRNAs that are expressed in the cells, preserve epithelial phenotypes and function as EMT suppressors. The levels of miR-200c were much higher in lung epithelial cells than in endothelial or mesenchymal cells in saline treated mice (Figure 2D). In contrast to miR-21, miR-200c decreased significantly in epithelial cells, implying that miR-200c also functions as an EMT suppressor in lung epithelial cells (Figure 2D).

**Figure 3:**
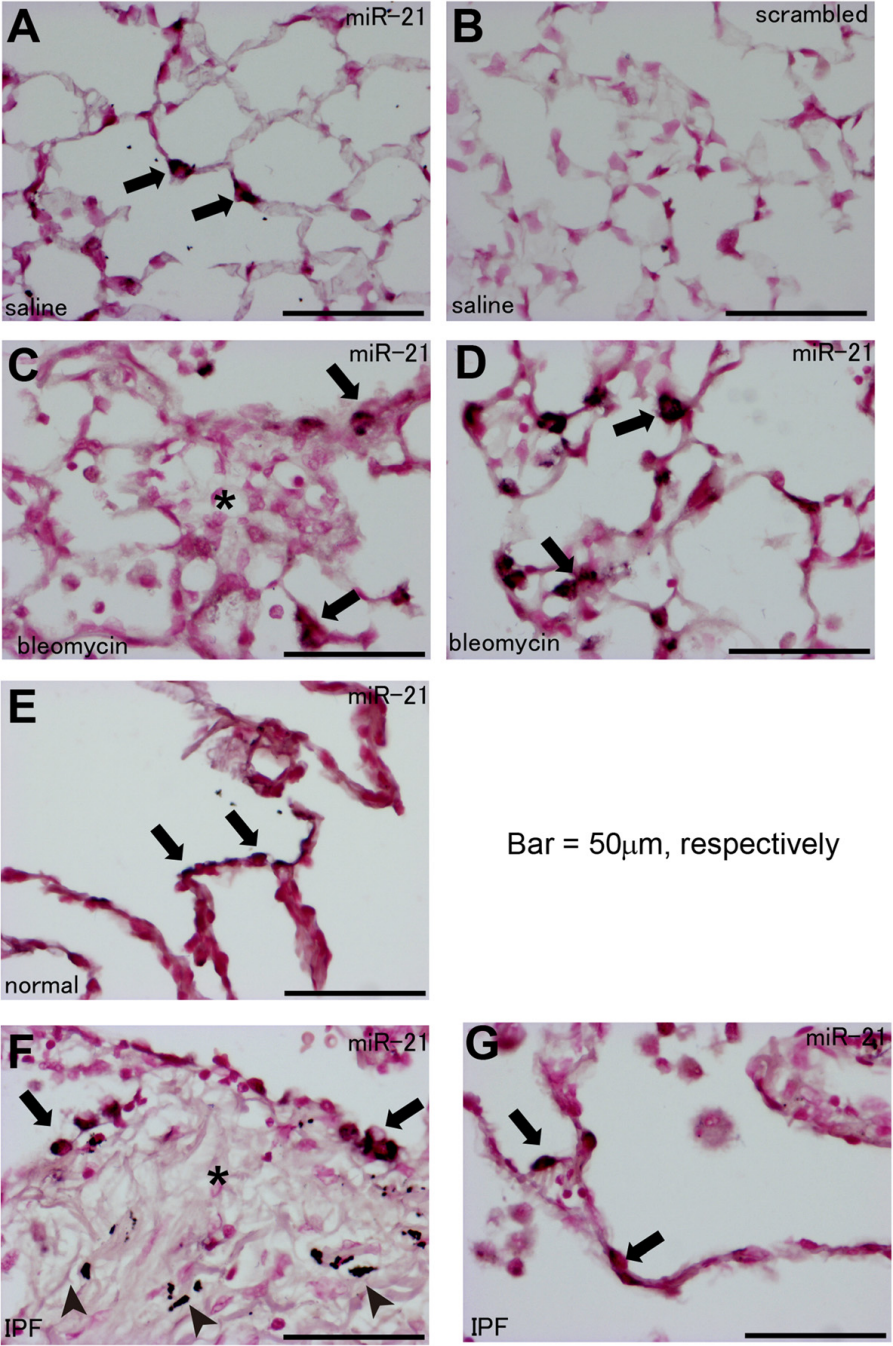
**Histological analyses for miR-21 expression in mouse and human lung tissues using in situ hybridization. (A-D)** In situ hybridization for miR-21 was performed in mouse lung tissues obtained from saline- **(A)** or bleomycin-treated mice **(C, D)**. Figure 3C and 3D show the areas demonstrating severe and mild fibrotic lesions, respectively. Staining using control probes with a scrambled sequence showed minimal background staining **(B)**. **(E-G)** In situ hybridization for miR-21 was performed in non-fibrotic lung tissue obtained from patients without fibrotic lung disease **(E)** and patients with IPF **(F, G)**. Figure 3F and 3G show the areas demonstrating severe and mild fibrotic lesions, respectively. Arrows indicate staining for miR-21. Asterisks indicate fibrotic foci. Arrowheads indicate anthracosis. Bar = 50 μm.

We also performed in situ hybridization for miR-21 in lung tissues from saline- or bleomycin-treated mice, and we observed scattered staining for miR-21 in the alveoli of saline-treated mouse lungs (Figure 3A). In particular, the morphology of the stained cells seemed to be compatible with that of alveolar type II cells (Figure 3A). As a control, background staining using control probes with a scrambled sequence was very weak (Figure 3B). In bleomycin-treated mice, staining for miR-21 was observed in cells surrounding fibrotic foci but not in cells within fibrotic foci (Figure 3C). Furthermore, increased staining for miR-21was observed in alveoli from areas showing milder fibrotic changes (Figure 3D), and the morphology of these stained cells was similar to that of alveolar type II cells (Figure 3D).

### miR-21 is increased in lung epithelial cells from patients with IPF

Our data showed that miR-21 expression is increased in lung epithelial cells during mouse experimental lung fibrosis. Therefore, we further examined the expression levels of miR-21 in the lungs of patients with IPF compared to that in non-fibrotic lungs. In situ hybridization for miR-21 showed scattered staining for miR-21 in the alveolar cells of non-fibrotic lung tissue (Figure 3E). In IPF lung tissues, staining for miR-21 was observed in cells surrounding fibrotic foci, but not in cells within fibrotic foci (Figure 3F). Staining for miR-21 was also observed in alveoli located in areas showing milder fibrotic changes (Figure 3G).

**Figure 4:**
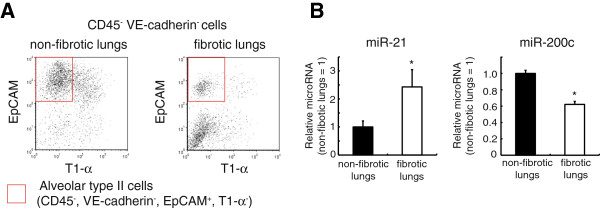
**miR-21 is increased in lung alveolar type II cells from idiopathic pulmonary fibrosis (IPF) patients. (A)** Representative flow cytometric analyses of CD45^-^ VE-cadherin^-^ lung cells for the isolation of human alveolar type II cells (CD45^-^ VE-cadherin^-^ EpCAM^+^ T1-α^-^ cells) from the non-fibrotic lungs of patients without fibrotic lung diseases and the fibrotic lungs of patients with IPF. **(B)** The relative expression of microRNAs is shown. The values represent the mean ± SEM (n = 3). The values relative to those of the human alveolar type II cells from non-fibrotic lungs are presented. The data were analyzed with Student’s t-test. * p < 0.05 versus the non-fibrotic lung group.

To investigate the changes in miR-21 expression in human lung epithelial cells from patients with IPF more precisely, we isolated alveolar type II cells from human lung specimens by flow cytometric cell sorting (Figure 4A) [20]. miR-21 expression was significantly higher in alveolar type II cells isolated from the lungs of IPF patients than in those present in non-fibrotic lungs (Figure 4B). In contrast, the levels of miR-200c were decreased significantly in alveolar type II cells from IPF patients (Figure 4B).

### The culture conditions that induce EMT by TGF-β up-regulated the expression of miR-21 in cultured alveolar epithelial cells

**Figure 5:**
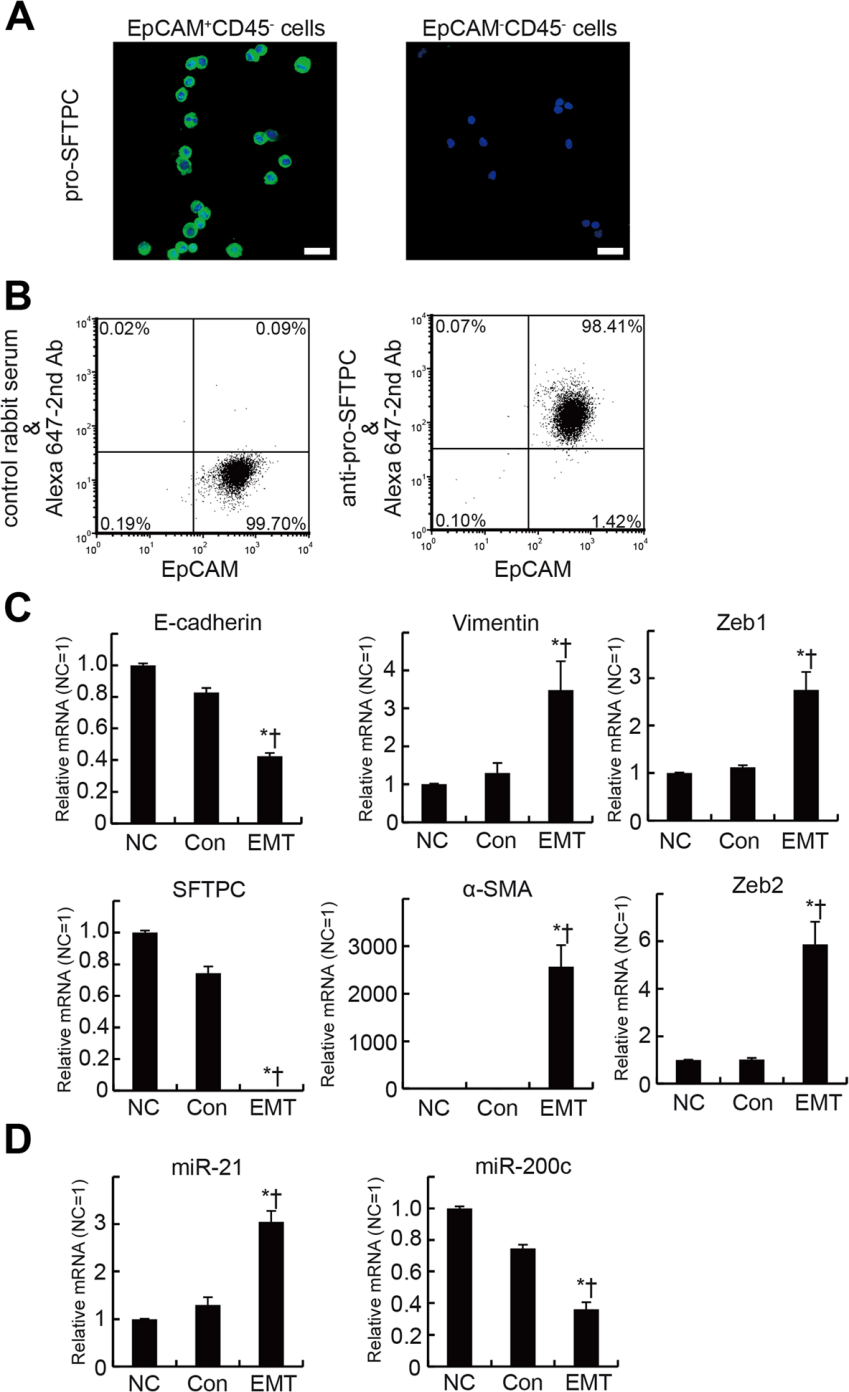
**The EMT-inducing culture conditions increase miR-21 expression in mouse lung alveolar epithelial cells. (A)** Representative flow cytometric analyses for the expression of pro-SFTPC in isolated mouse alveolar type 2 cells. Note that most of the isolated cells were positive for both EpCAM and pro-SFTPC. **(B)** Representative immunofluorescence staining for pro-SFTPC in the isolated cells. Most of the isolated cells were positive for pro-SFTPC (left), whereas EPCAM-CD45- cells were negative for pro-SFTPC (right). Scale bars: 20 μm. **(C)** The EMT-inducing culture conditions (EMT; cultured on fibronectin with TGF-β for 6 days) decrease lung epithelial cell marker gene expression and increase EMT marker gene expression in mouse lung alveolar epithelial cells. The relative expression of mRNA of lung epithelial cell marker genes (E-cadherin and SFTPC) or EMT marker genes (vimentin, α-SMA, Zeb1 and Zeb2) is shown. The values are mean ± SEM (n = 6). mRNA is expressed relative to levels observed prior to culture. The data were analyzed by one-way analysis of variance with a post hoc test (Scheffé’s test). * p < 0.05 versus cells not cultured (NC). † p < 0.05 versus control cultures (Con; cultured on Matrigel/collagen for 6 days). **(D)** The relative expression of microRNAs is shown. The values are mean ± SEM (n = 6). microRNA expression was relative to the levels observed prior to culture. The data were analyzed by one-way analysis of variance with a post hoc test (Scheffé’s test). * p < 0.05 versus NC. † p < 0.05 versus Con.

We observed an increase of miR-21 in isolated lung epithelial cells in which EMT occurred *in vivo* during bleomycin-induced lung injury. To clarify whether culture condition that induce EMT also increased miR-21 in alveolar epithelial type II cells cultured *in vitro*, we cultured mouse alveolar epithelial type II cells and induced EMT using endogenous and exogenous TGF-β as previously reported [23,24]. For cultured mouse alveolar type II cells, lung cell suspensions were prepared by intratracheal instillation of Dispase and agarose, followed by mechanical disaggregation of the lungs according to the method of Corti et al. [21]. CD45^+^ cells were depleted and then EpCAM^+^ cells were positively selected for using a magnetic beads-based cell sorting instead of flow cytometric cell sorting because of the high number of isolated cells required for *in vitro* culture experiments. We isolated 3.0 ± 0.5 million cells per mouse and >96% of the isolated cells were positive for pro-SFTPC as shown by intracellular immunostaining (Figure 5A) as well as by flow cytometric analyses (Figure 5B), indicating that the purity of the isolated alveolar type II cells is acceptable for *in vitro* culture experiments. The control culture conditions that can maintain the mouse type II cell phenotype *in vitro*[22] did not change the levels of expressions of mRNAs of E-cadherin, SFTPC, vimentin, α-SMA or ZEB1/2 when compared with those in freshly isolated alveolar type II cells (Figure 5C). The expression of E-cadherin and SFTPC decreased in alveolar type II cells cultured under EMT-inducing conditions, whereas the expression of vimentin, α-SMA and ZEB1/2 increased, suggesting that EMT had occurred in the cultured alveolar type II cells (Figure 5C).

We then examined the expression changes of miR-21 in cultured alveolar type II cells. The control culture conditions did not influence the expression of miR-21. The culture conditions that induced EMT by TGF-β significantly increased miR-21 expression in alveolar epithelial type II cells when compared with the freshly isolated cells and the cells cultured under the control conditions (Figure 5D). In contrast to miR-21, the EMT inducing culture conditions decreased the expression of miR-200c (Figure 5D). These observations indicated that miR-21 increased in pulmonary epithelial cells both *in vivo* and *in vitro* when EMT had occurred in those cells.

### The inhibition of miR-21 attenuates TGF-β-induced epithelial-mesenchymal transition in mouse alveolar type II cells

**Figure 6:**
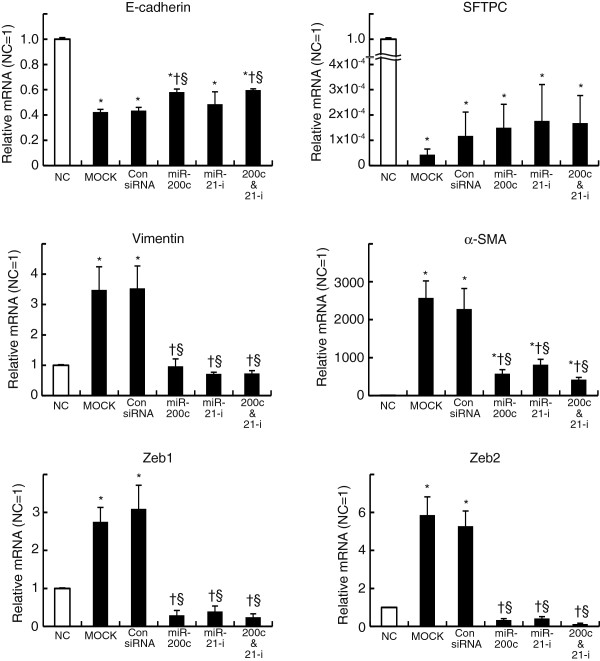
**miR-21 attenuates TGF-β-induced epithelial-mesenchymal transition in mouse alveolar type II cells.** The relative expression of mRNA of lung epithelial cell marker genes (E-cadherin and SFTPC) or EMT marker genes (vimentin, α-SMA, Zeb1 and Zeb2) prior to culture or 6 days after culture in the condition inducing EMT is shown. The values are mean ± SEM (n = 6). mRNA is expressed relative to levels observed prior to culture. NC: cells not cultured. MOCK: cells only treated with transfection reagents. Con siRNA: cells transfected with non-targeting siRNA. miR-200c: cells transfected with synthesized miR-200c. miR-21-i: cells transfected with miR-21 inhibitor. miR-200c & miR-21-i: cells co-transfected with both synthesized miR-200c and miR-21-i. The data were analyzed by one-way analysis of variance with a post hoc test (Scheffé’s test). * p < 0.05 versus NC. † p < 0.05 versus MOCK. § p < 0.05 versus Con siRNA.

**Figure 7:**
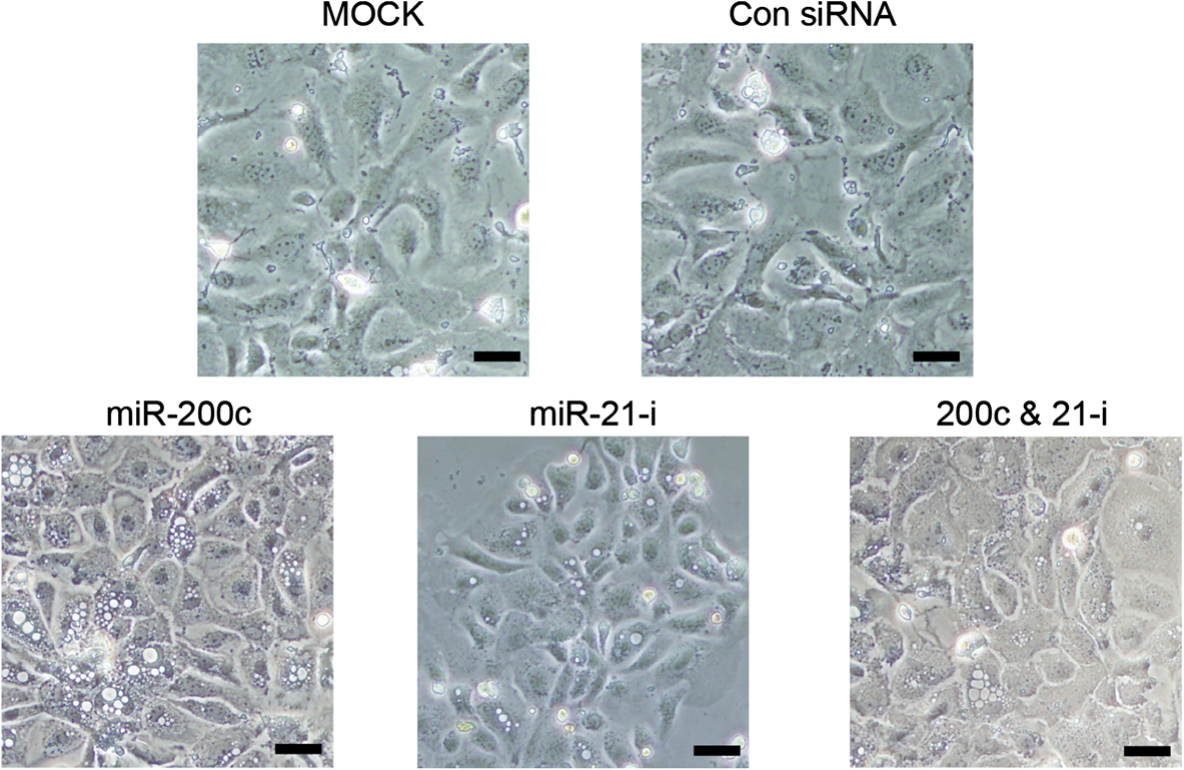
**Representative phase contrast images of mouse alveolar type II cells cultured under EMT-inducing conditions.** Images of the cells 6 days after culture are shown. MOCK: cells only treated with transfection reagents. Con siRNA: cells transfected with non-targeting siRNA. miR-200c: cells transfected with synthesized miR-200c. miR-21-i: cells transfected with miR-21 inhibitor. 200c & 21-i: cells co-transfected with both synthesized miR-200c and miR-21 inhibitor. Note that the cells cultured under EMT-inducing conditions adopted their spindle-like shape with irregular processes, whereas cells transfected with synthesized miR-200c and/or miR-21 inhibitor adopted a cobblestone-like appearance and had granules (lamellar bodies). Scale bars: 20 μm.

Our *in vivo* and *in vitro* experiments showed that miR-21 was upregulated in mouse lung epithelial cells during EMT. To clarify the contribution of miR-21 to EMT of lung alveolar epithelial cells, we examined whether the inhibition of miR-21attenuated EMT induced by TGF-β in primary mouse alveolar type II cells. A miR-21 inhibitor composed of single-stranded, modified RNAs that specifically inhibit miR-21 function was transfected into the cultured alveolar type II cells. We also transfected non-specific oligonucleotides as negative controls or synthesized miR-200c as positive controls because miR-200c has been reported to be an EMT suppressor. The transfection of synthesized miR-200c significantly attenuated the increased expression of vimentin and α-SMA as well as that of Zeb1/2 and prevented the decrease in E-cadherin expression in cultured mouse alveolar type II cells that was induced by the pro-EMT environment containing TGF-β (Figure 6). The miR-21 inhibitor also prevented expression of vimentin, α-SMA and ZEB1/2 but did not modify E-cadherin expression significantly, though we did observe the tendency of miR-21 to prevent the decrease in E-cadherin expression (Figure 6). Neither synthesized miR-200c nor the miR-21 inhibitor prevented the decrease in SFTPC expression. Transfection of both miR-200c and the miR-21 inhibitor had no significant added effect on the down-regulation of mesenchymal cell markers (Figure 6). Phase contrast images of cultured mouse alveolar type II cells showed that cells cultured under EMT-inducing conditions adopted a spindle-like shape with irregular processes, whereas cells transfected with miR-21 inhibitor as well as synthesized miR-200c adopted a cobblestone-like appearance and had lamellar body-like granules (Figure 7). These data showed that inhibition of miR-21 attenuated EMT induced by TGF-β in cultured primary mouse alveolar type II cells, suggesting the contribution of miR-21 to EMT in lung epithelial cells

## Discussion

Our study first demonstrated that miR-21 is up-regulated in isolated lung epithelial cells during bleomycin-induced lung injury as well as in cultured lung epithelial cells under EMT-inducing conditions. Inhibiting miR-21 prevented the EMT induced in cultured primary mouse alveolar type II cells, suggesting that miR-21 promotes EMT of lung epithelial cells.

Previous reports showed that miR-21 is increased in whole lung samples both in bleomycin-induced mouse models of lung fibrosis and in human patients with IPF [14-16,25]. The examinations by *in situ* hybridization and immunohistochemistry showed that myofibroblasts were the main source of the increased miR-21 levels during bleomycin-induced lung fibrosis [14,16]. miR-21 targets an inhibitory smad, SMAD7 and administration of miR-21 antisense probes attenuated the severity of bleomycin-induced fibrosis by blocking the positive feedback loop of TGF-β signaling [14]. The biological functions of miR-21 have also attracted the attention of researchers in various fields, including oncology. miR-21 is significantly upregulated in human cancer of various organs [13]. miR-21 targets the genes related to cell cycle control, apoptosis and invasion; thus, this microRNA behaves as an oncogenic factor [13]. Because most cancer cells are derived from epithelial cells in various tissues including lung, we hypothesized that miR-21 was also expressed and had a role in EMT of lung epithelial cells by promoting TGF-β signaling during lung fibrosis. We observed that miR-21 was expressed more in epithelial cells when compared with mesenchymal cells and endothelial cells in mouse lungs and increased miR-21 expression was observed in lung epithelial cells in the lungs of mice with bleomycin-induced fibrosis as well as the lungs of patients with IPF. Inhibition of miR-21 prevents the increase of mesenchymal markers and ZEB factors in cultured lung alveolar epithelial cells under the EMT-inducing conditions promoted by TGF-β. Our findings suggested that miR-21 works as a potential inducer of EMT in lung epithelial cells by promoting TGF-β signaling.

In situ hybridization revealed that miR-21 was upregulated in the fibrotic lungs of both mouse and human, as previously reported [14,25]. However, we observed that miR-21 was expressed in alveolar cells morphologically resembling alveolar type II cells in the normal lung. In addition, miR-21 expression was increased in cells surrounding fibrotic foci, rather than in the fibrotic foci themselves, during lung fibrosis, which is in contrast to previously published data showing by *in situ* hybridization and immunostaining that miR-21 is mainly expressed in myofibroblasts [14,25]. We developed a flow-cytometric cell sorting method for isolating epithelial, mesenchymal and endothelial cells from mouse and human lung tissue [26], because the expression patterns of molecules, including microRNAs, differ between cell types. These data showed that each isolated cell type expressed a specific set of gene markers and that miR-21 was more highly expressed in lung epithelial cells than in mesenchymal cells, in agreement with our in situ hybridization data. Therefore, we believe that we have carefully analyzed the expression of miR-21 in each lung cell type, including epithelial cells, and that our results reveal that miR-21 is expressed most prominently in lung epithelial cells. In contrast, previous reports did not perform precise analyses in specific cell types using expression analyses of whole lung samples, *in situ* hybridization and immunostaining. Similar to our report of the method for isolating cellular components from human lung tissue [26], techniques that can isolate specific cell types in the lung are useful for investigating the specific roles of a variety of molecules, including microRNAs, in specific cells under both physiological and pathological conditions.

The signaling pathways that are activated by transforming growth factor-β (TGF-β) are involved in promoting EMT [9,10,27]. TGF-β signaling induces the expression of transcription factors such as ZEB1, ZEB2, slug and snail, which negatively regulate the expression of E-cadherin and activate genes of mesenchymal markers such as vimentin and α-smooth muscle actin (α-SMA). Therefore, it is likely that microRNAs, including let-7d, miR-21 and miR200c, that are involved in TGF-β signaling also play a significant role in EMT during lung fibrosis [2,11,14].

It has also been reported that epithelial cells are potential sources of myofibroblasts via the epithelial-mesenchymal transition (EMT) in renal [28,29], hepatic [30,31] and lung fibrosis [23,24]. In contrast, a recent study using mouse genetic tools to track the fates of lung epithelial cells showed that both alveolar type II cells and Scgb1a1-positive lung cells are not a major source of myofibroblasts through EMT [32], suggesting that EMT does not directly contribute to lung fibrosis in vivo. Although the in vivo inhibition of miR-21 prevents bleomycin-induced lung fibrosis in mice [14], it remains debatable whether the inhibition of EMT improves or worsens the pathological condition of lung fibrosis.

## Conclusions

In summary, we demonstrated that miR-21 is increased mainly in lung epithelial cells in a mouse model of lung fibrosis as well as in isolated alveolar type II cells cultured under EMT-inducing conditions. Inhibiting the function of miR-21 resulted in the prevention of EMT induced by TGF-β. Taken together, our data suggest that miR-21 promotes EMT in lung epithelial cells during lung fibrosis.

## Abbreviations

α-SMA: Alpha smooth muscle actin; EMT: Epithelial-mesenchymal transition; IPF: Idiopathic pulmonary fibrosis; miR: microRNA; TGF: Transforming growth factor.

## Competing interests

The authors declare that they have no competing interests.

## Authors’ contributions

MY: Designed and performed experiments, analyzed data, produced graphs and tables, wrote manuscript; HK: designed and performed analyses, and contributed to writing of the manuscript; CO, TT, YT, TS, NF and MT: performed the experiments and analyzed data; KT and TS: performed in situ hybridization; IM: supervised the study. All authors read and approved the final manuscript.
